# Love, fear, and the human-animal bond: On adversity and multispecies relationships

**DOI:** 10.1016/j.cpnec.2021.100071

**Published:** 2021-07-07

**Authors:** Jennifer W. Applebaum, Evan L. MacLean, Shelby E. McDonald

**Affiliations:** a University of Florida, Department of Sociology and Criminology & Law, Gainesville, FL, USA; b University of Arizona, School of Anthropology & College of Veterinary Medicine, Tucson, AZ, USA; c Virginia Commonwealth University, School of Social Work, Richmond, VA, USA

**Keywords:** Human-animal interaction, Adversity, Multispecies families, Pets, Companion animals, Human-animal bond

## Abstract

Love and strong social bonds are known buffers in the experience of adversity. Humans often form strong bonds with non-human animals. The human-animal bond refers to a mutually beneficial and dynamic relationship between humans and non-human animals. Previous research suggests that strong bonds with pets may promote resilience in the experience of adversity, but a strong bond with a pet can also complicate this very experience of adversity, particularly among low-resourced and disadvantaged populations. What is the role of the human-animal bond in adversity, and what is the role of adversity in the bond between a human and a non-human animal? In this article we outline the state of research on the role of various types and sources of adversities in multispecies households (i.e., families, relationships) to consider this overarching question. We focus specifically on intimate partner violence, housing discrimination, LGBTQ+ identity-based discrimination, racism, neighborhood disadvantage, and economic inequality. We then outline an agenda for future research about love, adversity, and multispecies relationships, and discuss implications for public policy and community-based interventions.

## 1. Introduction

In contemporary society, love comes in many forms, including attachment bonds between people and their pets.^1^ Evidence of our close bonds and kinship with other species manifests in many ways. Particularly notable in the United States is the prevalence of cohabitation with pets and growing recognition of the modern, multispecies household.^2^ Indeed, it is estimated that approximately 60% of people in the U.S. live with a pet, a majority of which consider their pet(s) to be a member of the family [1,2]. Dogs and cats are the most prevalent animals kept as pets in the U.S., residing in approximately 46% and 25% of homes, respectively [1]. Adults’ social and emotional relationships with pets are often akin to a parental relationship with a child, whereas pets may serve as sibling figures for children [3–8]. In this vein, there is a growing movement away from anthropocentric views of family systems and toward an increasing recognition of multispecies families and households [9,10].

### 1.1. Overview

**Fig. 1.**
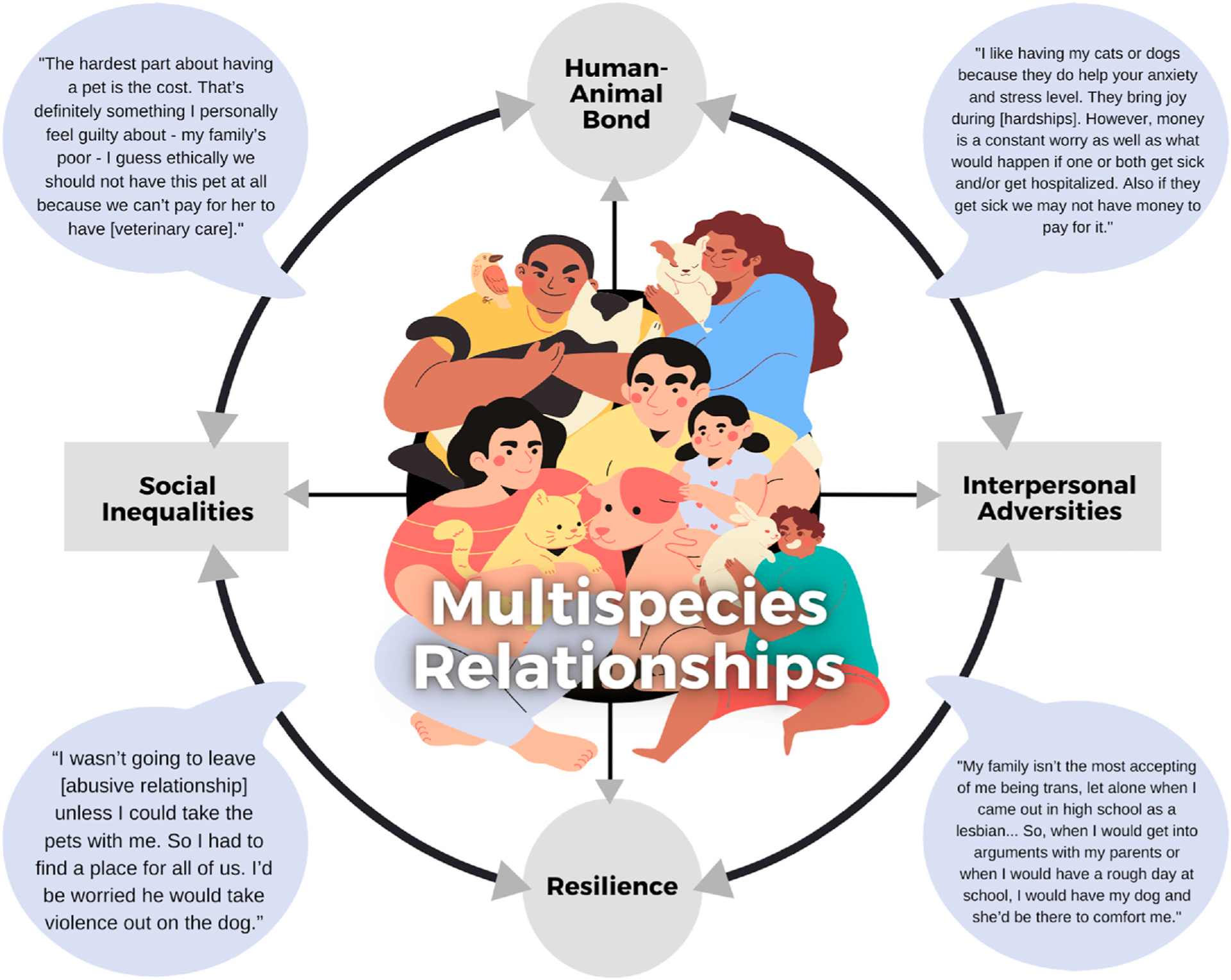
Conceptual relationships between the human-animal bond, interpersonal adversities, resilience, and social inequalities as they impact multispecies relationships. Quotes to illustrate these relationships are excerpts from the authors’ qualitative research.

Love and strong social bonds are known buffers in the experience of adversity [11–16]. However, the literature to date has failed to adequately consider how love that is characterized by the bond between a human and non-human animal (i.e., pet) impacts the lived experience of adversity. There is some evidence that strong bonds with pets may buffer stress and promote resilience in adverse social contexts. However, strong bonds with pets can also complicate adverse situations, and create barriers to meeting the social, emotional, and basic needs of both the individual and their pet. Our overarching argument is centered around the assertion that adversity interacts with the human-animal bond to create a complex interplay of disadvantage and resilience (see Fig. 1 for a visual representation). In this article we focus specifically on relationships between people and pets. We do not address issues concerning service animals, emotional support animals, or other working animals, though we recognize that individuals often form strong emotional bonds with working animals. Importantly, in some cases the issues we discuss below will also apply to working animals when the line between working animal and pet may blur (e.g., when the handler of a service dog develops an emotional attachment with the dog, see [17]).

The purpose of this essay is threefold: (1) first, we review current theoretical orientations toward explaining relationships between humans and non-human animals. We follow with a working definition, and explanation of our orientation toward the construct of adversity. (2) Next, we review select literature concerning human-pet relationships and adversities in many forms. (3) We set an agenda for future research with the goal of further understanding the myriad ways in which adverse experiences and scenarios may impact people, pets, and their shared bonds within multispecies relationships. We conclude with recommendations for public policy and community partnerships aimed at alleviating some of the adversities faced by multispecies families.

## 2. Theoretical orientations toward explaining the human-animal bond

A driving factor of the prevalence of multispecies families is the human-animal bond (HAB), a term defined as the, “mutually beneficial and dynamic relationship between people and other animals that is influenced by behaviors that are essential to the health and wellbeing of both” [18]. It is hypothesized that human-pet dynamics satisfy needs in both humans and animals for companionship, emotional support, nurturing and love [19–21]. However, the reciprocal nature of this dynamic is most applicable to relationships with pets that share mammalian social cognition and emotion. The neural and anatomical systems that serve as mechanisms of positive sociality are shared among humans and their most preferred domestic species (e.g., dogs), and likely permit the development of strong HABs [22,23]. In addition to this evolutionary perspective, multiple theoretical orientations have been applied to understand the love people have for their pets. Attachment and social support theory, in particular, are often applied as frameworks for identifying underlying biobehavioral and social mechanisms through which interactions with pets may be beneficial [24,25]. Broadly, the application of these theories emphasize that humans and animals develop social bonds (attachment) and that relationships with animals provide indirect (facilitation of human interaction) and direct (e.g., positive regard and companionship) forms of social support to humans [25–29] (see Ref. [30] for a review). It is well documented that, across the lifespan, interactions with pets provide their human companions with a sense of social support that mirrors that of attachment bonds with other people, yet offers unique characteristics that diverge from the complex dynamics of human interactions.

One of the unique characteristics of human-animal relationships is that pets are often perceived by humans as being a reliable, nonjudgmental source of companionship and support, especially in the context of stressful situations or adversity [31–34]. As a result, children and adults often seek out interactions with their pets in times of stress if the animal provides a sense of comfort, consistency, or safety. It is hypothesized that the benefits of human-animal interaction (HAI), a term often used to refer to any situation (e.g., interactions with household pets, interactions with therapy animals) where there is contact between humans and animals [18], may be most pronounced when individuals are in a “stress state” [22], which makes human-animal relationships particularly important to consider when evaluating vulnerability and resilience to adverse experiences.

At a biological level, bonds between humans and pets are hypothesized to rely on neuroendocrine pathways involving oxytocin and vasopressin, molecules that play critical roles in mammalian emotions and social behavior. In the brain, these neuropeptides act as neuro-transmitters and neuromodulators, with important actions in limbic regions and the autonomic nervous system [35]. Both peptides are also released peripherally, where they act as hormones and provide feedback to the central nervous system. Although previous research has focused largely on prosocial functions of oxytocin, both oxytocin and vasopressin play important (and sometimes opposite) roles in stress and fear, making them highly relevant for our understanding of biological responses to adversity in the context of HABs.

Pendry and Vandagriff provide an important framework from which to understand the biobehavioral mechanisms through which HAI attenuates the stress response system [36]. Building on prior research, they proposed the HAI-HPA (Hypothalamic–pituitary–adrenal axis) Transactional Model which posits that the socio-emotional support provided by pets buffers the stress response both prior to and after activation of the stress response system. They suggest that this ultimately disrupts the association between stress and concomitant psychological maladjustment. Pendry and Vandagriff emphasize that the presence of an animal may assist people in perceiving potential stressors as less threatening [36]. This can, in some circumstances, prevent the activation of the stress response system completely. Although Pendry and Vandagriff focus on cortisol release as an end product of the HPA axis, it is important to note that HPA activity can be significantly attenuated by oxytocin, or stimulated by vasopressin, through their actions in the central nervous system [37–39]. Given their hypothesized roles in HAIs, both oxytocin and vasopressin may be key mediators of stress physiology in this context. Although current evidence for this hypothesis remains limited, several studies report increased oxytocin and/or decreased cortisol or vasopressin concentrations after affiliative contact between people and pets [40–43]. The HAI-HPA framework also posits that once the stress response system is activated, pets may support humans in re-appraising whether their situation is still stressful. This cognitive reappraisal, together with increased social contact with nonhuman animals may further dampen physiological arousal through social buffering [30,36,43–46].

### 2.1. HABs in the context of adversity

The evolutionary and theoretical perspectives outlined above offer insights into the importance of humans’ social bonds with non-human animals in the context of adversity. In this article, we use the term adversity to refer to adverse life experiences (hardships, challenges, misfortunes) that have the potential to influence human development in a way that disrupts typical development, compromises an individual’s adjustment, and/or has the potential to lead to undesirable outcomes [47,48]. Adversity can involve single forms of acute or chronic stress. However, given that adversity is typically experienced as multiple events, rather than a single experience, it most often involves a combination of acute and chronic stressors, some of which are preventable or malleable and others that are not. Among forms of adversity commonly studied, poverty, household dysfunction (e.g., exposure to domestic violence, substance use), psychological, physical, and sexual forms of abuse, neighborhood dysfunction (e.g., neighborhood violence, crime), and experiences of racial and/or ethnic discrimination and other forms of minority stress are considered to have particularly harmful impacts on short- and long-term adjustment (e.g., psychological and physical health) [49–53].

Adversity increases an individual’s risk for a variety of negative health and social outcomes, such as poor psychological and physical health outcomes and housing and economic insecurity [49–53]. Broadly, it is argued that dysregulation of the stress response system is a common mechanism underlying links between adversity (particularly early life adversity) and poor health outcomes. Specifically, this occurs via impacts on the HPA axis and autonomic nervous system, including developmental programming of the oxytocin and glucocorticoid systems [54,55]. These biological consequences include, but are not limited to, compromised neuroendocrine functioning (through alterations in patterns of release or epigenetic modifications to receptors), inflammation, and dysregulation of the immune system [56,57]. The ways in which an individual’s physiological system responds to changing environmental demands, such as adverse experiences, produces changes that may be adaptive short-term, but maladaptive at later periods of development or in other contexts. Specifically, from a functional perspective, stress responses mobilize energy reserves facilitating one’s ability to mount a defense to a threat. Though highly adaptive in the short term, chronic stress diverts energetic resources required for growth, digestion, and repair, leading to life history tradeoffs with deleterious consequences in the long term [58,59].

In addition, adversity may influence the characteristics of the HAB. For example, selective social behavior and strong bonds with pets may form in response to adversity and the perception that one’s welfare is dependent on the presence of the other [22]. Positive social relationships and social support have particular value in the context of adversity; the ability to manage stress is inextricably linked with social behavior and engagement [22]. Indeed, it is hypothesized that HAI may potentially reverse some of the harmful impacts of chronic stress by contributing to functional increases in oxytocin or its receptor [22,60, 61].

Despite rapid growth in research and theory regarding the HAB, and benefits of HAI, few studies have considered how HABs contribute to negative emotions, such as fear and stress responses, and how this may impact the stress response system, particularly among populations disproportionately impacted by adversity. Many people live in social environments (e.g., family and neighborhood contexts) that increase their risk of exposure to maladaptive and atypical forms of HAI. In these situations, individuals may experience fear as a result of being exposed to potentially traumatic experiences (e.g., violent households) or adverse situations (e.g., poverty) that threaten an individual’s ability to maintain a relationship with a beloved pet and/or threaten the welfare of an animal to which an individual is bonded. Given that HAI involves complex, dynamic emotions and behaviors, it is critical that research consider the interplay between positive and negative experiences associated with the HAB, and the ways that multispecies relationships impact, and are affected by, adversity. From a biological perspective, adversity is known to have long-lasting effects on many of the same pathways implicated in the HAB [62,63]. However, biological responses under conditions of adversity may be opposite to those associated with the protective effects of sociality. For example, responses to chronic adversity may include upregulation of vasopressin or its receptors, facilitating defensive behavior, or together with oxytocin, selective social bonds [63]. Thus, both the basal patterns of these neuroendocrine systems, as well as their acute responses to HAI, may differ markedly in people experiencing chronic adversity.

## 3. What we currently know about the interplay of adversity and multispecies relationships

In this section we review literature on the interactions between adversity and multispecies bonds, and the resulting impact on people and pets within these relationships. We pay specific attention to the ways that love and fear interact within these multispecies households (i.e., families, relationships) to result in greatly varied health and well-being outcomes for all household members, including both human and non-human animals. We recognize there are myriad sources and contributors to adversity, as well as responses to them. These adversities range from interpersonal trauma to systemic inequalities, which can interact and accumulate to result in extremely varied and individualized experiences. Additionally, we recognize that individuals and groups are often represented by various social categorizations, which can combine for varied experiences of disadvantage or privilege (see: intersectionality [64]). Although we do not specifically consider intersectionality in this paper, it is implied at times throughout. In the interest of limiting our scope, we have chosen to focus the below literature review on the following topics: intimate partner violence, housing discrimination, LGBTQ+ identity-based discrimination, racism, neighborhood disadvantage, and economic inequality.

### 3.1. Intimate partner violence

Few areas of research demonstrate the intersection of love, fear, and the HAB as effectively as studies on the link between intimate partner violence (IPV) and animal cruelty. IPV is a form of family violence characterized by a variety of behaviors within an intimate relationship that are intended to assert control and power over another individual. These behaviors include psychological, physical, and/or sexual harm by a current or former intimate partner [65–67]. IPV occurs among all forms of contemporary intimate relationships. However, in this section we highlight studies of the statistically more prevalent, and more frequently reported, scenario of a woman being victimized by a male partner. Nearly 24% of women in the United States will experience IPV during their lifetime [68] during which they may experience numerous tactics of coercive domination and retaliation, including a partner’s intentional harm or threat to harm animals as a form of psychological abuse of them, their pet, and/or their children [31,65,66,69–74].

Across studies, it is estimated that 25%–71% of IPV survivors with pets report having experienced violence toward an animal by their abusive partner [75,76]. Faver and Cavazos surveyed women receiving domestic violence shelter services and found that among women who did not report maltreatment of pets by their partner, 51% indicated their pet was an important source of support. In contrast, 88% of participants who reported animal maltreatment by their partner identified the mal-treated pet as a “very important” source of emotional support [77]. Results of this study and others suggest that a victim’s love for their pet (s) may be a prominent factor in violent perpetrators’ motivation to engage in animal cruelty [31]. Adding to the psychological burden of experiencing IPV and concomitant animal cruelty, adult victims often witness their child(ren)’s abuse of pets in the home. Children who are exposed to animal cruelty are more likely to engage in aggressive and cruel behaviors towards pets, making the emotional experience of being subject to IPV behaviors and concomitant animal cruelty even more psychologically burdensome and traumatic for adult victims who also care for children.

Numerous qualitative studies suggest that adult and child survivors of IPV live within a duality of finding support in their bond with a pet while also experiencing chronic fear and guilt about having that bond exploited by their partner [31,78]. Victims of IPV are often socially isolated from friends and family, and for both youth and adults an animal companion may be their only form of consistent and reliable emotional support and stress-reduction [33,79]. The close bonds that arise between people and their pets in psychologically and physically abusive living situations may lead adult and child survivors to become engaged (physically, verbally, as a means to protect their pet) in incidents of animal cruelty, which may increase their risk of physical injury and/or death by an abusive partner [31–33,80]. Moreover, in the absence of access to pet-sheltering or pet-fostering services, pet-friendly alternative housing, and/or financial resources, victims of IPV may choose to maintain their relationship or living situation with their abuser out of fear for their pet and what might happen to the animal in their absence [31]. This compromises the safety of all victimized members of the family, including beloved pets.

### 3.2. Housing discrimination

Access to affordable housing in the U.S. is a widespread problem. It is estimated that, as of 2020, over 50% of renters in the U.S. were considered “rent burdened,” which is defined as spending more than 30% of monthly income on rent [81]. Housing insecurity has only worsened during the COVID-19 pandemic, as an estimated 30–40 million renters are facing eviction in 2021 [82]. While some municipalities have placed restrictions on the amount of extra monthly rent a landlord can officially charge for allowing a pet on the property, these ordinances are uncommon. Moreover, pet ownership does not qualify as a protected status or identity under the Fair Housing Act, therefore, there are no sweeping regulations nor policies that protect multispecies families from discrimination on the basis of including a non-human animal. Policies restricting the number, type, and size of family pets in rental units and condominium associations are extremely common. In 2005 Carlisle-Frank, Frank, and Nielsen estimated that only 9% of the rental housing stock in the U.S. was “pet-friendly” in that it had no restrictions on any pet-related factors, while 44% had limited pet allowance, and 47% allowed no pets at all. The study, along with a similar study in 2018, found that pet-friendly rental units had higher average rent and higher average deposits than properties that did not allow pets, and pet-owning renters often settled for lower-quality housing in less desirable locations [83,84].

The above issues are reflected in recent studies: pet ownership is a common barrier to finding and maintaining affordable rental housing both within the U.S. and elsewhere. The experience of renting with pets can itself cause perceived feelings of insecurity and instability, sometimes even prompting renters to hide pets from landlords, thereby putting themselves at an increased risk of eviction if the pet is discovered [85]. The effects of lower-quality, limited availability, and higher price tags for rental pet-friendly rental housing, compared to properties that restrict pets, also contribute to internalized feelings of instability among renters [86]. Pet owners with more resources have the option to be selective in rental properties, while those with less resources are often forced to accept lower-quality housing or avoid contacting the property manager for repairs for fear of being considered a nuisance (regardless of the involvement of the pet in the issue) [86].

The problem of renting with pets is especially salient for families and individuals who are already facing other forms of disadvantage, such as discrimination and/or resource constraint. Rental units that restrict pets, or certain types of pets, are more common in disadvantaged communities and communities of color [87]. Additionally, pet policies are not regulated within supported housing for older adults, such as assisted living facilities or nursing homes, and those who wish to age-in-place (i.e., in communities versus supported housing) with their pets may also find it difficult to find appropriate housing. Toohey and Rock [88] illustrate the need for what they call “more-than-human solidarity” in housing policy in order to support economically vulnerable older adults in multispecies families. They found that older adults would often risk their own health and wellbeing in order to preserve the relationship with their pet(s). In some cases, pet owners who cannot find affordable housing that can accommodate their pet(s) are forced to choose homelessness in order to avoid having to relinquish, re-home, or abandon their pet(s) [89]. Housing issues are a frequently-cited reason for shelter relinquishment of pets [90]. Further, those who do find themselves unable to access housing due to pet ownership (and/or other issues) may also find their pets prevent them from entering sheltered housing or accessing health and social services [91]. This can pose a particularly difficult situation as pets are known to be important supports and motivators for those in very precarious situations, such as people who are unhoused [92,93]. Pets are often cited as a source of resilience and motivation for maintaining health for those facing substantial hardships, such as homelessness [93], a diagnosis of HIV [94], and identity-based discrimination, upon which we elaborate below.

### 3.3. LGBTQ+ identity-based discrimination

The dynamic interplay of love, fear, and HABs is also demonstrated in literature on HAI in marginalized populations, such as historically (and currently) underrepresented groups (e.g., racialized minority populations, sexual and gender minority populations, etc.). However, few studies have examined how the experiences of risk and resilience associated with living with pets may be impacted by the unique stressors and sociocultural context faced by individuals who hold marginalized identities [34]. In this section, we review the emerging literature on HAI among sexual and gender minority groups (e.g., lesbian, gay, bisexual, transgender, queer, and other marginalized sexual and gender identities, or “LGBTQ^+^”) to demonstrate additional ways in which love for pets and multispecies relationships are shaped by adversity. We also highlight ways in which HAI may operate as a risk and protective factor in the context of sexual and gender minority stress.

Emerging evidence suggests that the risks and benefits of living with pets may be particularly salient for LGBTQ+ individuals. LGBTQ+ communities experience disproportionate risk for adversity (e.g., employment discrimination, housing insecurity, family and peer rejection) and a broad range of health disparities which stem from oppressive sociocultural structures and attitudes toward LGBTQ+ people; the experience of navigating cis-heteronormative social contexts and associated stressors is often called minority stress [34,95–97]. Minority stress broadly includes varied adverse experiences that occur in overt and covert forms, such as discrimination, victimization, social rejection, and internalized stigma [98,99]. The accumulation of minority stress contributes to LGBTQ+ youth and adults’ increased risk for a broad range of physical (e.g., obesity [100]), behavioral (e.g., substance use, risky behaviors [101]), and mental health (e.g., internalizing behavior symptoms, suicidal ideation [102,103]) disparities. Such outcomes are inextricably linked with other outcomes of oppression, such high rates of housing instability and economic insecurity in this population [104–107].

Social support (quality and number of confidants) and belongingness are important factors that promote healthy identity development and resilience in this population [108–112] as well as known buffers of the association between adversity and mental health problems [113–115]. Studies indicate that pets are frequently considered to be “chosen family” and confidants among LGBTQ+ individuals [116]. Furthermore, several studies link pet ownership and other aspects of HAI with resilience and positive coping in this population. Both pet ownership and positive engagement with pets have been identified as factors that mitigate associations between familial victimization and psychological stress in studies of LGBTQ+ populations [117,118]. Moreover, a recent study of LGBTQ+ emerging adults found an indirect effect of exposure to LGBTQ+ microaggressions on personal hardiness (an indicator of interpersonal resilience) via HAI [119]. Specifically, microaggressions were associated with increases in HAI (as measured by comfort from and attachment to pets); in turn, increases in HAI were associated with higher levels of personal hardiness among these youth [119]. Another study of LGBTQ+ emerging adults found that the effect of identity-based victimization on self-esteem was moderated by the degree to which participants sought out comfort from pets, such that victimization was not related to decreases in self-esteem when participants reported moderate to high levels of comfort from pets [120]. Such findings have been mirrored in recent qualitative work that found nearly 74% of 117 LGBTQ+ young adults who lived with pets reported that their pet was an important form of support that helped them positively cope with minority stress. In addition, youth reported that pets helped to promote social capital and facilitate healthy interactions with family members, peers, and new acquaintances [34].

Despite these benefits of HAI, the subtle and overt forms of minority stress that are disproportionately prevalent in the daily lives of LGBTQ+ people may make this population more vulnerable to potential hardships associated with pet ownership. The aforementioned qualitative study of 117 LGBTQ+ young adults also found that 90% of the sample reported stress associated with living with or caring for pets (behavioral problems, impact on expenses, impact on social relationships) and that these stressors were salient and influential experiences that compromised, or had the potential to compromise, wellbeing via emotional and financial burdens [34]. In that study, more than 60% of the sample recounted caregiver burden associated with meeting pets’ needs, such as medical and behavioral health issues, being able to secure an alternative caregiver in emergency situations, and having difficulty managing finances as a result. More than half also described psychological stress associated with loss, potential loss, and/or harm to their pet; some even disclosed ruminating of when their pet would die, and feared if they would be able to cope and adjust to that life transition. This form of stress is particularly concerning among populations, such LGBTQ+ communities, that experience increased risk for psychological stress and maladjustment, barriers to affirming healthcare, and an increased likelihood of experiencing poverty and reduced social support [121,122].

Although pets may be an important source of social support and companionship that can promote resilience in the context of adversity, multispecies families experience unique challenges that have the potential to exacerbate vulnerabilities that result from systemic inequalities. The degree to which bonds with pets may exacerbate vulnerability, particularly in the absence of essential resources is discussed further below. Next, we discuss the degree to which comfort and coping through HAI may be compromised by another form of adversity—racism.

### 3.4. Racism

We begin this section with a statement regarding our own positionality: the authors of this essay identify as White, therefore, we acknowledge that we cannot fully appreciate nor understand the experience of pet ownership for people of color.^3^ That said, we feel it is necessary to draw attention to these ongoing issues of social injustice as they are inherently related to love, fear, and the HAB. Issues of housing and economic inequalities, which we cover in other sections, are also deeply and inextricably linked to historical and ongoing marginalization on the basis of race and ethnicity. Here we discuss other salient examples of chronic and acute adversities borne out of racism as they relate to the HAB: the history of dogs as a tool of oppression, current discriminatory practices in animal welfare, and social control via animal-related trauma and violence. We focus our attention primarily on anti-Black racism, though we acknowledge that racism against other racial and ethnic groups is also a driver of inequities in the experience of multispecies relationships.

Race is a known predictor of pet ownership in the U.S.: according to recent population estimates, approximately 29% of Black individuals in the U.S. own pets, compared to over 70% of White individuals [1]. Some may point to cultural differences to explain this disparity. Although we agree that culture certainly plays a role in patterns of pet-keeping (see [123]), here we discuss other possible explanations for this disparity including deep roots in historical and systemic anti-Black racism, specifically within the U.S. In discussing pet ownership among Black families and communities in the U.S., one must first acknowledge the ways in which dogs have been used as an historical tool of racial oppression (see Refs. [124–126] for in-depth discussions of these topics). Dogs were used by slave owners to intimidate, control, and even kill Black people in the American south during the era of slavery. The legacy continued into the civil rights era when dogs were routinely used by police to violently quell public demonstrations protesting racial segregation [126]. In fact, these patterns persist in contemporary society. For example, in a 2015 report by the U.S. Department of Justice following the police killing of Michael Brown found the Ferguson, Missouri police department had a notable “… pattern of deploying canines to bite individuals when the articulated facts do not justify this significant use of force” (p. 31), and that every victim of the police dog incidents were Black [127]. There were also reports of police dog intimidation during the Black Lives Matter protests in Ferguson as well as the uprisings in 2020. In using the term “culture” to explain low rates of pet ownership among Black individuals in the U.S., one is inherently (though perhaps unknowingly) invoking this violent, racist history. This history is also inherently linked to the current demographic patterns of pet ownership, as well as ongoing barriers to pet ownership for people of color.

The continued oppression and discrimination against people of color is evident in current practices and assumptions within animal welfare, subsequently limiting access to pet ownership for these populations [128]. While pet ownership may be less common among Black individuals, compared to White individuals, to our knowledge there is no evidence for racial or ethnic differences in the ways that pets are related to and cared for, barring issues of economic resources and agency (e.g., access to veterinary care), among U.S. populations [129–131]. However, for many animal welfare and control organizations, race does factor into perceptions of who should own pets, or who is considered a “responsible pet owner” [128,132]. For example, Guenther [133] argues that pit bull type dogs are broadly conceptualized as companions to Black and Latinx men, which has been used by some to rationalize discrimination against owners of pit bulls (i.e., Breed Specific Legislation, “dangerous dog” clauses), as well as high rates of pit bull shelter euthanasia. Pit bulls in particular have been subject to ongoing misconceptions regarding their proclivities and traits. Some have argued this can be traced to their cultural association with men of color, and how racial bias and oppression may manifest via some animal welfare practices (see [134–136]).

Beyond breed-specific discrimination, communities of color experience disproportionate social control and punishment via institutionalized practices of animal control agencies, in addition to policing [132]. For example, Hawes and colleagues found that the enforcement of the city of Denver’s breed specific legislation (which banned pit bull type dogs from 1989 to 2020) was most likely to occur in geographic areas with notable racial tension between multiracial communities and those that were predominantly White [137]. Notably, the practice of disproportionate enforcement of animal control policies and breed bans can lead to the confiscation or forced relinquishment of pets from the very owners who may depend on them for emotional support due to their experiences of chronic adversity [128]. Relatedly, in another example of racialized trauma, Bloch and Martinez found that officer-involved shootings in Los Angeles that resulted in the death of a dog (i.e., lethal police shootings of dogs) were clustered in low-income communities of color [138]. Not only do communities of color experience disproportionate trauma via violence toward family pets, they are also subject to a disproportionate share of state violence from police dog attacks [139]. In the context of racism, pets (particularly dogs) can be both a source of trauma and adversity as administered by the oppressor, as well as a potential source of comfort and support for the oppressed. In an example of the latter, a qualitative study with a sample of 15 women of color from various racial and ethnic backgrounds (i.e., nine Latinx women, two Asian women, two Native American women, and two African American women) found evidence for a common theme of “reciprocity” between pets and owners [130]. The women of color in this study described mutually-beneficial and at times intuitive relationships with their pets, attending to one another’s needs and offering companionship [130].

### 3.5. Neighborhood disadvantage

A widely-cited study by Wood and colleagues outlines a potential mechanism for the ways in which pets may be beneficial to health: social capital [140]. Social capital is considered to be the extent to which an individual is connected and embedded within their communities and social networks, and is a robust predictor of physical and mental health outcomes [141]. Wood and colleagues found that, among their Australian sample, pet owners tended to report stronger and more frequent social connections within their neighborhood and community, which was considered to be connected to interactions involving pets. These findings were replicated a decade later in the U.S., leading the authors to once again conclude that pet ownership appeared to be a “conduit” to social capital, thereby benefiting the health and wellbeing of people in multispecies relationships [142]. Notably, while Wood and colleagues appear to have collected considerable demographic information from their respondents, they did not explore the potential moderating role of race on these associations. This may lead one to wonder, considering enduring racial housing segregation and the legacy of redlining: does the social capital effect of pet ownership extend beyond White individuals? Mayorga-Gallo argues that, while White residents in Durham, North Carolina, USA did experience social capital benefits of pet ownership, the non-White residents of the same community did not, and in fact routinely experienced negative pet-related interactions with their White neighbors [143]. The very social connectedness experienced by White pet owners was used as a tool to draw racial and ethnic boundaries and maintain racial distance in their multiracial community [143].

Spatial context, dubbed “neighborhood effects” in social science, refers to the geographic contextual and ecological factors that influence the social lives of the individuals located within the geospatial bounds [144]. This concept has been extended to human-animal relationships to show that contextual factors determined by spatial analysis influence the chances of a pet being separated from its family and ultimately admitted to the shelter. For example, Ly et al. showed that contextual, spatial factors such as quality of housing, economic disadvantage, and unemployment (among other factors) predicted patterns of shelter relinquishment from various neighborhoods in the Vancouver area [145]. This was also reflected by Spencer and colleagues, who found that spatial patterns of child maltreatment were related to spatial patterns of animal intake to a municipal shelter in a community in Florida [146]. Both studies point to geographic patterns of social inequities, concentrated in areas of disadvantage, as significant risk factors for disrupting the HAB and disadvantaging people and pets in multispecies households.

### 3.6. Economic inequality

Economic inequality shapes the health and wellbeing of all members of multispecies households. Among marginalized and disadvantaged people, the responsibility of pet ownership, and the experience of a bond with a pet, may compete with other priorities for limited resources. This phenomenon creates an extra vulnerability in the face of adversities. Previous studies suggest that economically disadvantaged pet owners will be more likely than other pet owners to risk their own health and safety in order to prioritize their pet(s), particularly when the individual has a strong bond to their pet (e.g. Refs. [91,93,147]).

Stoltz and colleagues show that dogs have become actors within the “budgetary unit” of American families, therefore the family decision-makers are responsible for considering the dog’s wants and needs alongside that of human family members [148]. They assert that non-human animals kept as companions, and in particular dogs, have shifted in American society to be conceptualized as actors who consume with people, whose wants and needs are included in the resource budgeting of the household [148]. Pets, due to their liminal status of family/property, as well as animal control laws and policies, are vulnerable and wholly dependent upon human caretakers throughout the entirety of their lives. If their guardian fails to care for them, they are usually subject to impoundment or abandonment, and often untimely death via euthanasia (in the case of sheltering) or neglect (if abandoned). This could lead economically vulnerable pet owners to make choices that may appear irrational, such as allowing their own health and wellbeing to suffer, while prioritizing the health, welfare, perceived wants, and/or needs of their pets. These patterns are reflected in responses to emergencies and disasters: bonded pet owners are at a higher risk of failing to evacuate their homes if they are unable to bring their pet(s) with them, as owners who have less resources at their disposal are also less likely to have access to pet-friendly hotels or friends or family who can temporarily offer their family shelter [149–151]. The public health emergency posed by COVID-19 complicated healthcare planning for pet owners, as some reported they would not be able to seek emergency care out of concern that contingency accommodation for their pets might not be available; this fear was especially salient for pet owners who had limited socioeconomic resources, and those who reported a strong attachment bond to their pet [147].

Guenther outlines the concept of “the irresponsible owner,” which Guenther dubs a myth, in The Lives and Deaths of Shelter Animals [128]. Guenther posits that the construct of responsible pet ownership permeates animal welfare and sheltering. This in turn places individual responsibility upon pet owners for the (alleged) insufficient care of pets, which often results in shelter relinquishment or field intakes by animal control officers when pets are found free-roaming in communities. Guenther’s main argument centers around this misplaced blame and subsequent labeling of whole populations (characterized often by race/ethnicity and/or socioeconomic status) as “irresponsible” and therefore unworthy of the companionship of a dog or cat. Guenther offers an alternative explanation: the mechanisms of social control, oppression, and structural inequality place economically disadvantaged (and usually Black and/or Latinx) pet owners in a state of precarity, thereby subjecting them to acute and chronic adversity (e.g., housing instability, deportation, involvement in the justice system) that often results in the forced separation of pet and (human) family, regardless of how much they wish to preserve that relationship [128]. Notably, economic inequalities are rarely considered in HAI research to date. We discuss this in more detail, as well as other directions for future research, in the following section.

## 4. An agenda for future research

Researchers interested in adversity and multispecies bonds face a multidisciplinary field with somewhat disjointed methodological and theoretical traditions. Here we detail three topics we feel warrant additional research, and make recommendations for future directions. We build upon the current state of research outlined in previous sections to investigate the interplay between adversity and the HAB.

### 4.1. The impact of pets on human wellbeing: better understanding the role of adversity

The field of HAI has been wrestling with an ongoing question for some time: are pets good for human health and wellbeing? Currently, the thrust of the overarching question is in the realm of *for whom*, *under what conditions*, and also, *why for some and not others*? We posit that, in order to get at these questions, researchers must better understand the role of adversity in multispecies relationships. Here we recommend a few avenues of potential exploration. First, we urge HAI researchers to direct efforts toward better understanding HABs in marginalized populations with the goal of exploring the interaction of stressors and supports associated with HAI. It is our belief that a false assumption has been made in considering the effects of HAI to be comparable for all people. This assumption fails to recognize the unique effects of how identity and HAI play out in everyday life. Notably, we are unaware of any research concerning the potential moderating role of comfort from pets on the relationship between experiences of racial or ethnic discrimination and wellbeing among people of color. This topic warrants investigation. Second, there are myriad validated measures concerning constructs like attachment to pets and comfort from pets (see [152]) however, the field needs better tools for measuring the negative aspects of HAI and the HAB. Notably, the “Perceived Costs” subscale from the Monash Dog Owner Relationship Scale (MDORS [153]) is useful for a generalized measurement of some of the potential drawbacks of dog ownership, specifically. However, the MDORS lacks specificity with respect to understanding myriad potential pet-related stressors and is not generalizable to other types of pets. For example, a quantitative measure of pet-related stress reflecting themes in recent qualitative studies, such as issues related to caring for pets with behavioral issues, or difficulties balancing pet caregiving with other priorities and responsibilities (see [154–157]) would be useful for survey researchers interested in a better understanding of the entire experience of pet ownership. Third, theoretical models that aim to identify the mechanisms through which HAI benefits human health and wellbeing primarily focus on the benefits of touch and support in downregulating physiological reactions to stress. However, we argue that the generalizability of these theories will remain limited if chronic and acute stressors related to the HAB are not accounted for in studies that test these frameworks, particularly in the context of studying relationships with household pets. Currently we know little about how adversity interacts with the physiological systems that are hypothesized as central to the HAB. However, preliminary studies of animal-assisted interventions in populations that have suffered from trauma reveal effects that are sometimes opposite to those in other populations [158]. Given that many neuroendocrine pathways implicated in HAI can also be modified by trauma or chronic stress [54,159], it is reasonable to expect that biological responses to HAI will vary significantly between populations. Thus, studies with convenience samples may fail to adequately capture key psychological and physiological features of the HAB in the context of adversity.

### 4.2. Demographic patterns of pet ownership: uncovering mechanisms related to adversity

As we discussed above, population estimates of the demographic correlates to pet ownership in the U.S. show that non-Latinx White individuals tend to own pets at much higher rates than do people of color, particularly Black individuals [1]. Future research should further interrogate why this disparity exists, particularly if pets may be health-promoting for those experiencing adversity and trauma. For example, we currently have a very limited understanding of the (un) availability of pet-friendly rental housing (for analyses in North Carolina see [87]; Edmonton see [160]) and the racial and socioeconomic distribution of these properties. However, a more comprehensive, nation-wide analysis is necessary for understanding the larger picture of the barriers of pet ownership to low-income and historically marginalized individuals. Additionally, more research related to HAI and racial and ethnic discrimination is warranted, both regarding anti-Black racism, as well as other minoritized groups. Researchers may consider integrating the concept of intersectionality in these endeavors (see [64]). Researchers should also consider how the species of pet may factor into these relationships, as the hardships and barriers experienced for dog owners may be fundamentally different from those experienced by reptile owners, for example. Similarly, different species might differ in their potential to be beneficial for social support or stress buffering in the experience of hardship and adversity.

Animal welfare organizations such as the Humane Society of the United States (HSUS) and the American Society for the Prevention of Cruelty to Animals (ASPCA) have made great strides in the provision of veterinary care to vulnerable communities with the goal of empowering pet owners to maintain the health and welfare of their pets, and also prevent pet relinquishment to shelters. However, questions remain about the lasting impact of these programs, which are often provided on a temporary basis and, due to funding limitations, are not permanent fixtures in their communities. Veterinary care is often expensive; low-income, and even “middle class” pet owners often cannot afford to provide their pets with regular healthcare that is recommended by the veterinary industry. Thinking beyond questions of access and affordability, as veterinary medicine (and the pet product industry) is a business, researchers may find themselves wondering if the concept of idealized pet health maintenance and welfare (e.g., the utility of goods and services beyond basic health and welfare) is serving pets and their owners. We might ask: does the conceptualization of optimal pet health and welfare unintentionally reproduce racial and socioeconomic inequalities in access to pet ownership? If so, how might we better support marginalized and disadvantaged individuals in ways that serve both pet and owner?

### 4.3. Data to investigate adversity and multispecies relationships

The availability of data is integral to research efforts for understanding the role of adversity in human-pet relationships, and more broadly, interactions between pets and people in society. We argue for the inclusion of extensive HAI measures in large, probability-based data collection efforts that allow for generalization to entire populations. Because HAI research has long relied on less rigorous sampling methodology (and quite often nonprobability sampling), entire populations, particularly those who are non-White and/or low-SES, are not represented in the current HAI knowledge base. Along these lines, although rigor in HAI has improved in recent years, there is a crucial need for further longitudinal HAI research, as well as better understanding of pet relationships beyond the simple dichotomy of pet owner versus non-pet owner. Short HAI measures have been included in a handful of large population surveys, such as the General Social Survey in 2018, the Health and Retirement Study in 2012, and the Panel Study of Income Dynamics’s Child Development Supplement in 2014 and 2019. However, these HAI measures were overly simplistic and failed to capture the complexity of HABs and HAIs. While they did allow for the investigation of some outstanding HAI questions, they were not administered to full samples, often had a limited availability of correlates due to data structure and survey scope, and had not undergone rigorous psychometric evaluations in diverse populations [1].

Beyond gathering nationally representative data on HAI, careful attention must be paid to study design concerning marginalized and disadvantaged (i.e., “vulnerable”) populations. For example, HAI researchers may consider methodology such as participatory action research, which both involves the research participants in the process, as well as empowers the research subjects to put the findings into action in both practice and policy [161]. Researchers may also consider partnering with community organizations that provide services to disadvantaged populations in order to assess their clients’ pet-related needs. Further, simply bringing research findings to the attention of community stakeholders could encourage organizations to take needed steps toward supporting people and pets together.

## 5. Conclusion

In this essay we explored the interplay between love and fear as they impact multispecies households. We focused on literature concerning several types of adversities as examples of the ways in which adversity and the HAB intersect within and between multispecies families. Specifically, we discussed the ways in which intimate partner violence, housing discrimination, LGBTQ+ identity-based discrimination, racism, neighborhood disadvantage, and economic inequality each impact, and are impacted by, the HAB. We also focused on the ways in which these adversities can simultaneously strengthen and challenge the HAB, and suggested that our knowledge of the physiological mechanisms involved in HABs will remain limited without future studies focusing on marginalized populations and experiences of adversity. We continue here with a brief discussion of two limitations in this article and conclude with recommendations for public policy aimed at supporting people and pets in multispecies relationships.

### 5.1. Limitations

We acknowledge three major limitations in this article. First, the majority of evidence we discuss pertains specifically to “Western” societies. Future research should consider the role of adversity on human-animal relationships in other parts of the world that we failed to consider in this article, in particular: non-Western nations. Second, while we consider animal welfare and shelter outcomes in this article, a majority of the content is anthropocentric, that is, prioritizing the perspectives and experiences of humans above those of non-human animals. Much of HAI research suffers from this limitation (see [162,163]) and we assert future research should consider non-human animal experiences in addition to those of humans. Third, we do not discuss adversity and HAB as it relates to relationships between humans and service animals, emotional support animals, or other working animals. We acknowledge there are indeed hardships and adversities for individuals with disabilities who have relationships with these types of animals (e.g. Ref. [164]) and therefore we recommend future research further consider those experiences and perspectives.

### 5.2. Policy implications

Opportunities exist for public policy aimed at alleviating adversities placed on multispecies relationships. First, we recommend the inclusion of pet ownership as a protected status against housing discrimination. As we detail above, many of the barriers faced by multispecies families that in turn force the separation of pets from their people originate in the inability to find affordable rental housing and temporary shelter services that allow pets. We predict that sweeping pet-friendly housing policy will make enormous strides in preserving multispecies bonds, particularly among marginalized communities and those who experience disproportionate adversity. Additionally, communities should encourage the partnering of human social welfare services with those of animal services in order to provide support to the holistic family unit in times of hardship and adversity. For example, the provision of temporary boarding or foster services for pets of individuals who are temporarily unavailable to care for them due to hospitalization or other issues would prevent both the permanent separation of pets from their families, as well as alleviate barriers to entering in-patient healthcare or accessing other services (see Refs. [165–169] for discussions on the impact of these types of interventions). Models for these services exist in various communities and can be replicated,^4^ but not without community support and financial resources. Moving toward a model of support for the holistic family unit, inclusive of pets, is necessary for the mitigation of adversities and the promotion of the HAB.

## Footnotes

1 In the interest of brevity and consistency, we chose to use the term “pet” throughout this essay to refer to a non-human animal that lives alongside a human or humans. This term is synonymous with the term “companion animal.”

2 In this essay we use the terms “multispecies household,” “multispecies family,” “multispecies bond,” and “multispecies relationship” interchangeably to refer to the unit characterized by the relationship or bond between a human (or humans) and a non-human animal (or animals) who typically coexist together. We use these terms to refer to the family or household unit, though “household” does not necessarily refer to those contained within a house (i.e., we include unhoused individuals and their animal companions in these descriptions), and “family” does not necessarily refer to more than one individual and one animal, nor does it necessarily refer to those who would be recognized legally as family.

3 We use the term “people of color” per the American Psychological Association guidelines (see: https://apastyle.apa.org/style-grammar-guidelines/bias-free-language/racial-ethnic-minorities) to refer to groups or populations who do not identify as White. We acknowledge there are inherent issues and disagreements with terminology, and our hope is our language choice is such that it will cause the least harm to marginalized individuals.

4 See https://www.humananimalsupportservices.org/ for examples and tool-kits for integrating these types of services into existing community programs.
