# Erratum regarding missing Declaration of competing interest statements in previously published article

**DOI:** 10.1016/j.cpnec.2022.100112

**Published:** 2022-01-20

Declaration of Competing Interest statements were not included in the published version of the following article that appeared in the August 2021 issue of Comprehensive Psychoneuroendocrinology.

The appropriate Declaration/Competing Interest statements, provided by the Authors, are included below.

“Love, fear, and the human-animal bond: On adversity and multispecies relationships” (Comprehensive Psychoneuroendocrinology, 2021; 7C: 100071) 10.1016/j.cpnec.2021.100071. Declaration of competing interest: The Authors have no interests to declare.

